# Predictive Relationships Between Death Anxiety and Fear of Cancer Recurrence in Patients with Breast Cancer: A Cross-Lagged Panel Network Analysis

**DOI:** 10.3390/curroncol32120685

**Published:** 2025-12-03

**Authors:** Furong Chen, Ying Xiong, Siyu Li, Qihan Zhang, Yiguo Deng, Zhirui Xiao, M. Tish Knobf, Zengjie Ye

**Affiliations:** 1 School of Nursing, Guangzhou Medical University, Guangzhou 511436, China; cfrllufy@stu.gzhmu.edu.cn (F.C.); 2024211707@stu.gzhmu.edu.cn (S.L.); 2024211693@stu.gzhmu.edu.cn (Q.Z.); yiguo@stu.gzhmu.edu.cn (Y.D.); 2 School of Nursing, Guangzhou University of Chinese Medicine, Guangzhou 510405, China; 20231120409@stu.gzucm.edu.cn; 3 School of Nursing, University of South China, Hengyang 421001, China; 20222015111457@stu.usc.edu.cn; 4 School of Nursing, Yale University, Orange, CT 06477, USA

**Keywords:** breast cancer patients, death anxiety, fear of cancer recurrence, cross-lagged panel network analyses, psycho-oncology

## Abstract

The aim of this study was to explore the longitudinal relationship between death anxiety (DA) and fear of cancer recurrence (FCR) in women newly diagnosed with breast cancer at baseline and 3 months post-discharge. A total of 426 women with breast cancer completed the Templer’s Death Anxiety Scale and the Fear of Cancer Recurrence Inventory at hospital discharge and 3 months later. Cross-lagged panel analysis (CLPA) was used to describe the relationship of the two variables (DA and FCR) over time and identify the optimal intervention symptom nodes for breast cancer patients in different stages. The findings suggest that the specific symptoms of DA, known as “cognition”, predict the subsequent symptom development for a variety of mental health problems in the network structure. The “Psychological distress” symptom in FCR is the most susceptible to other symptoms. In addition, death-related cognition may be a bridge symptom that connects the co-occurrence of DA and FCR. Death-related “time awareness” is the optimal symptom node for intervention in early-stage breast cancer patients, while it is “cognition” in advanced patients. The death-related cognition and emotional regulation of death may be the best target for interventions among breast cancer patients, considering their DA coincides with FCR. The best intervention for patients with early-stage breast cancer may be the time awareness of death, while it may be more effective for patients with advanced cancer to be educated about disease and death, as well as to enhance correct perception.

## Simple Summary

Women diagnosed with breast cancer often experience two deeply interconnected psychological challenges: anxiety about death and fear that their cancer will return. However, we do not fully understand how these fears develop over time or which one leads to the other. In this study, we followed 426 women with newly diagnosed breast cancer for three months to understand the relationship between these two types of fear. We found that thoughts about death tend to come first and then trigger fears about cancer returning. Specifically, how patients think about death and time plays a crucial role in connecting these two fears. Importantly, these results suggest that the best way to help patients depends on their cancer stage: for early-stage patients, helping them process their awareness of time and mortality is most beneficial, while for advanced-stage patients, addressing their thoughts and understanding about death and disease is more effective. These findings can help doctors and psychologists provide better, personalized mental health support for breast cancer patients at different stages of their journey.

## 1. Introduction

Women diagnosed and treated for breast cancer report a wide variety of physical and psychological symptoms. In contrast to the trajectory of physical symptoms related to primary treatment (surgery or breast-conserving treatment with radiotherapy) [1], psychological symptoms, such as death anxiety (DA), often persist over time and can be very distressing [2]. DA has been less frequently studied but has been documented in patients with a variety of cancers, including breast [3,4]. Females with cancer, and specifically women with breast cancer, report high death anxiety [4]. However, few studies have focused on the core symptoms of DA and its development in cancer patients.

Fear of cancer recurrence (FCR) is reported in at least half of women with breast cancer and negatively affects quality of life [5,6]. It is necessary to have further clarification in the longitudinal development of core symptoms of FCR among Chinese breast cancer patients. Although FCR and DA are qualitatively distinct, FCR often manifests as specific, cancer-related apprehensions about relapse or progression, while DA encompasses broader existential fears of mortality; they frequently co-occur and may amplify each other in cancer patients [7,8,9]. Although some FCR models note the potentially threatening aspects of cancer, such as general anxiety [10,11], death anxiety is not explicitly defined or mentioned in these frameworks. Curran [12] highlighted the importance of comprehending the essence of the fear that underlies anxiety associated with death in individuals suffering from cancer, and Simonelli et al.’s [7] conceptualization of FCR has placed greater emphasis on DA, suggesting that unaddressed existential fears may sustain recurrence-related anxiety over time. This interconnection implies that interventions targeting shared mechanisms, such as cognitive reappraisal of death-related thoughts, could disrupt symptom cascades and improve outcomes. In terms of practical investigations, several cross-sectional studies have confirmed that DA predicts FCR [8,9], whereas others have reported that predictors of elevated DA include FCR [13]. However, none of these current studies were able to infer a predictive relationship between DA and FCR explicitly [14], underscoring the need for longitudinal studies like ours to provide empirical evidence for directional links and inform targeted psycho-oncological strategies.

Network analysis has been a more common method used for psychological symptom networks in recent years. Researchers frequently utilized cross-sectional data to build non-directional networks; however, such networks lack the capability to reflect temporal dynamics and determine the causality or directionality within the relationships [15]. Therefore, longitudinal data are needed to model temporal relationships between symptoms. The cross-lagged panel network (CLPN) is an innovative approach that enables the application of network methods to longitudinal data to simultaneously test the complex relationships and longitudinal processes among all the symptoms in the network model [16]. Thus, we aimed to identify optimal intervention symptoms, defined as network nodes with high out-expected influence (OEI), which exert strong predictive effects on downstream symptoms and could serve as leverage points for disrupting maladaptive cycles in psycho-oncology.

Overall, while several of the aforementioned studies in this paper have contributed significant understanding into the symptomatic interconnections between DA and FCR, a fully integrated comprehension of these mechanisms is still restricted. Ignoring the evolving interplay among symptoms across time may have far-reaching consequences and hinder the effectiveness of targeted interventions. To bridge this gap, we aim to delve into three specific questions by utilizing the framework provided by network analysis modeling: (1) the optimal intervention symptoms of DA and FCR; (2) the co-occurrence and longitudinal development in the relationship between DA and FCR symptoms; (3) the network differences between early-stage and advanced-stage BC patients.

## 2. Methods

### 2.1. Participants

For cancer patients, death anxiety and fear of cancer recurrence are evident at different stages of their disease, perhaps at first diagnosis, at the time of discharge, and at each re-examination [3]. The Guidelines for breast cancer diagnosis and treatment by the China Anti-cancer Association (2024 edition) suggest that breast cancer should be followed up every 3 months for 2 years after surgery [17]. Therefore, we chose to conduct this survey when breast cancer patients were first discharged (T1) and 3 months after being discharged (T2).

From April to August 2024, we recruited 450 female breast cancer patients from the “Be Resilient to Breast Cancer (BRBC)” program, using convenience sampling. The BRBC is an ongoing observational project in China focused on assessing psychological resilience and related outcomes in female breast cancer survivors through longitudinal surveys and network analyses [18,19,20]. In this context, it serves as a recruitment platform for studies like ours, where participants complete standardized questionnaires at key time points (e.g., discharge and follow-up) without receiving any structured interventions, mentoring, or supportive-expressive components. This approach allows for naturalistic tracking of symptoms such as DA and FCR in a real-world cohort of newly diagnosed patients.

The inclusion criteria were as follows: (1) patients aged ≥18 years, (2) those diagnosed with breast cancer who have completed their first treatment and were ready to be discharged. The exclusion criteria included an inability to cooperate due to severe symptoms and patients with pre-existing mental health conditions, whether ongoing or historical, as well as individuals contending with serious ailments apart from their cancer diagnosis.

### 2.2. Sample Size

Cases with invalid data were excluded at T2, and a total of 426 questionnaires were validly answered with a completion rate of 94.67%, which were used for CLPN analysis. Based on the outcomes of the power analysis detailed in Supplementary Figure S1, with a sample size of n = 426, the impact of correlation, sensitivity, and specificity of the network model were all deemed satisfactory (each exceeding 0.6) [21].

### 2.3. Ethical Statement

The research protocol adheres to the ethical guidelines established by the University of South China (Approval No. 2022NHHL013). The gathered data, utilized solely for the purposes of this investigation, were maintained with rigorous confidentiality.

### 2.4. Measurements

#### 2.4.1. Basic Characteristics

Considering that previous studies involved much more recognized influencing factors of DA and FCR among cancer patients [3,13], we collected fundamental characteristics of the participants, which included age, level of education, marital status, occupation, and average monthly household income per person, to serve as covariates in our analysis.

#### 2.4.2. The Templer’s Death Anxiety Scale

Developed by Templer and colleagues [22], it consists of fifteen items, structured around five dimensions: cognition, emotion, time awareness, stress, and pain. An increased total score reflects a heightened degree of death anxiety. A score totaling 7 or more indicates the presence of death anxiety. The Cronbach α coefficient of the scale was 0.867, and the test–retest reliability was 0.831 [23]. We employed the Chinese adaptation of the scale [24], for which Cronbach’s α in this study was 0.88.

#### 2.4.3. The Fear of Cancer Recurrence Inventory

The Fear of Cancer Recurrence Inventory (FCRI) was regarded as one of the most robust instruments with sound psychometric properties for assessing FCR [25]. There are 42 items with seven dimensions: trigger factors, severity, psychological distress, functioning impairment, insight, seeking comfort, and coping strategies. A higher aggregate score indicates a greater level of FCR. The total Cronbach’s α was 0.96 [26]. The Cronbach’s *α* of this scale was 0.846 in our study.

### 2.5. Statistical Analysis

We utilized IBM SPSS v28.0 for descriptive statistics and R v4.4.0 to construct Cross-Lagged Panel Networks (CLPN).

#### 2.5.1. Network Analysis

Through a chain of regularized regressions and by utilizing penalized maximum likelihood estimation incorporating a LASSO penalty, we assessed the autoregressive and cross-lagged coefficients. These autoregressive pathways are depicted as the dominant associations in the network structure, visually downplaying the visibility of the cross-lagged relations [27]. Unlike SEM-based models, CLPN does not report global fit indices (e.g., Chi-square test, Root Mean Square Error of Approximation, Comparative Fit Index), emphasizing edge stability via nonparametric bootstrapping (1000 iterations) and correlation stability (CS) coefficients instead [16,21]. Therefore, the network diagram after removing the autoregressive association was shown in the main manuscript [16]. To examine the directional relationships between dimensions, estimations of In-Expected Influence (IEI) and Out-Expected Influence (OEI) were conducted [16,28]. Furthermore, the one-step bridge Expected Influence was calculated to pinpoint the bridging factors linking DA and FCR. The logistic regression coefficients, such as edge weights, were converted into odds ratios (ORs) by exponentiation. We utilized the glmnet package for the computation of regression analyses, while the plotting of all graphical representations was accomplished using the qgraph package [29,30].

#### 2.5.2. Post Hoc Stability and Accuracy Analysis

We calculated 95% confidence intervals (CIs) for each network via nonparametric bootstrapping (1000 bootstraps) to estimate the network stability [21]. The accuracy of network parameters was examined by computing the correlation stability (CS) coefficient. A CS coefficient above 0.25 is recommended, and values above 0.50 are indicative of high robustness [21].

#### 2.5.3. Network Comparison

A network comparison was carried out between early-stage (I and II) breast cancer patient networks and those of advanced-stage (III and IV) cancer patients to infer the changes in associations among domains of DA and FCR in different stages.

## 3. Results

### 3.1. Demographic Characteristics

A total of 24 individuals were discharged at T2 compared to T1, and there were no significant demographic differences between the dislodged and analyzed samples. Missing data at T2 (4.67%) were handled via listwise deletion in glmnet, suitable for low missing completely at random (MCAR) attrition (Little’s test: *χ*^2^ = 19.67, *p* = 0.15). The sample consisted of 426 patients with breast cancer, with ages ranging from 19 to 67 years (mean = 47.30, SD = 11.27). Other information was provided in Table 1.

### 3.2. Death Anxiety and Fear of Recurrence

Mean total scores for death anxiety were 8.25 (SD = 4.71) at T1 and 7.24 (SD = 4.35) at T2 with slight improvement. The average score of FCR was 79.64 (SD = 25.69) at T1, and this score lessened to 74.39 (SD = 22.63) at the subsequent evaluation (T2). Further specifics for DA and FCR were delineated in Table 2. The correlation analysis between DA and FCR at T1 was presented in Table S1.

### 3.3. Cross-Lagged Panel Network Models

**Figure 1:**
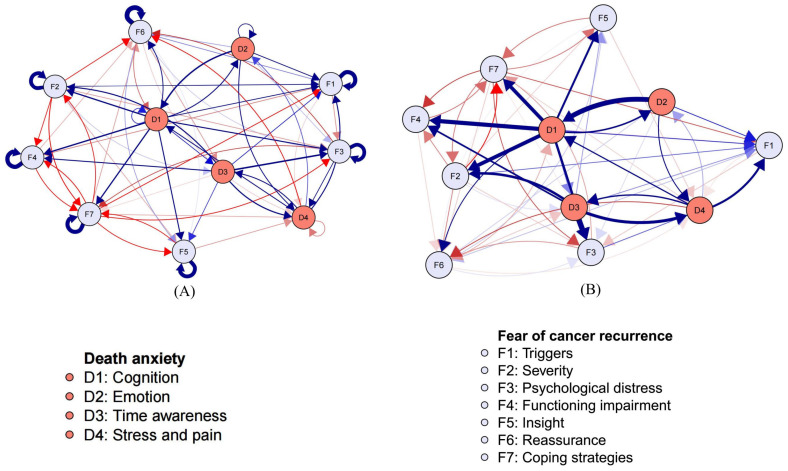
The cross-lagged panel networks. Figure 1 (**A**) includes all autoregressive and cross-lagging edges, and autoregressive edges and covariates were excluded in Figure 1 (**B**) for visual interpretation. Arrows represent unique longitudinal relationships. Blue edges indicate positive relationships (i.e., odds ratios greater than 1), and red edges indicate negative relationships (i.e., odds ratios less than 1). Edge thickness represents the strength of the odds ratio, such that thicker edges represent stronger relations.

**Figure 2:**
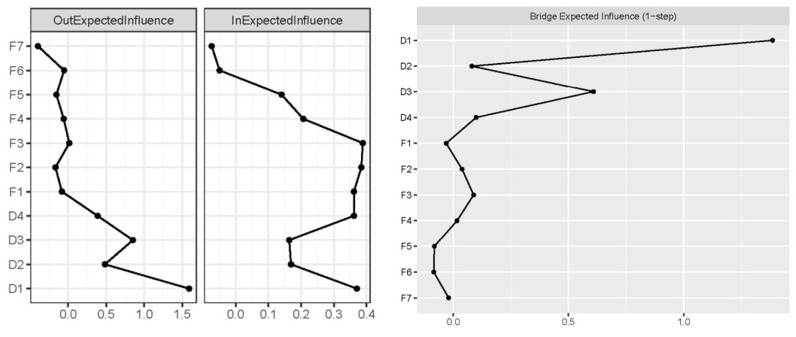
Centrality estimates in the T1 → T2 network. Larger values reflect greater centrality.

The cross-lagged network from hospital discharge to 3 months later was visualized as a directed network, with the inclusion of autoregressive edges, as shown in Figure 1. It was noteworthy that the autoregressive edges (mean OR = 1.10) were stronger than the cross-lagged ones (mean OR = 1.02). After achieving regularization convergence and eliminating the autoregressive paths, 60 non-zero cross-lagged edges were retained, with 33 of them (55.0%) being positive. The visual interpretability of the cross-lagged edges was shown in Figure 2.

Regarding the longitudinal dynamics within death anxiety, the two strongest cross-lagged edges were D2: Emotion → D1: Cognition (OR = 1.33) and D3: Time awareness → D4: Stress and pain (OR = 1.23). Concerning the longitudinal processes within fear of cancer recurrence, the two strongest edges were F2: Severity → F1: Triggers (OR = 1.06) and F1: Triggers → F6: Reassurance (OR = 1.03). With respect to the longitudinal impact between DA and FCR, the two foremost bridging edges were D3: Time awareness → F3: Psychological distress (OR = 1.36) and D1: Cognition → F2: Severity (OR = 1.27). Specific details were shown in Table 3.

The nodes demonstrating the greatest out-EI were D1: Cognition (OEI = 1.590) and D3: Time awareness (OEI = 0.851), signifying they yield considerable predictive capacity for other symptoms. On the other hand, F3: Psychological distress (IEI = 0.389) and F2: Severity (IEI = 0.384) were the nodes with the highest in-EI, denoting that they were substantially predicted by other symptoms in the network. The two strongest bridge-EI (1-step) nodes were D1: Cognition [BEI (1-step)] = 1.386) and D3: Time awareness [BEI (1-step) = 0.609]. The centrality estimates across the network were depicted in Figure 2.

### 3.4. Network Stability and Accuracy

The 95% bootstrapped confidence intervals (CIs) surrounding the edge weights were found to be relatively narrow to moderately sized (refer to Supplementary Figure S2), indicating that the network exhibited an acceptable level of stability. The precision of the network, as evaluated by in-EI (with a CS-coefficient of 0.439), out-EI (CS-coefficient of 0.594), and bridge-EI (CS-coefficient of 0.516), suggested that the network’s accuracy was moderate (see Supplementary Figure S3). The outcomes of edge weight difference tests and centrality difference tests were displayed in Supplementary Figures S4 and S5, respectively.

### 3.5. Network Comparison

**Figure 3:**
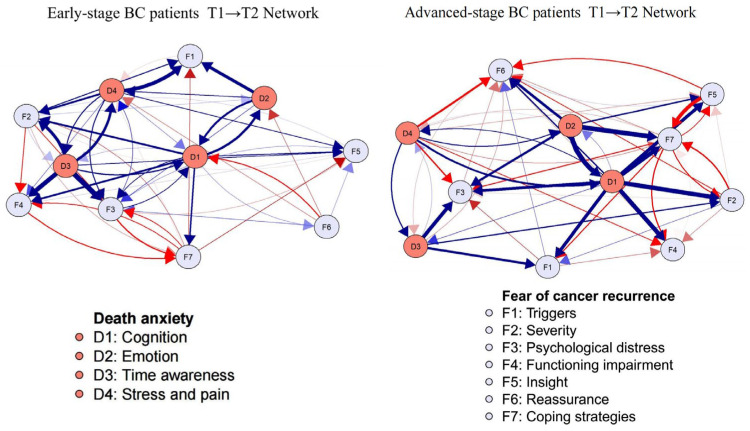
The comparison of T1 → T2 cross-lagged panel network among early-stage and advanced-stage BC patients.

**Figure 4:**
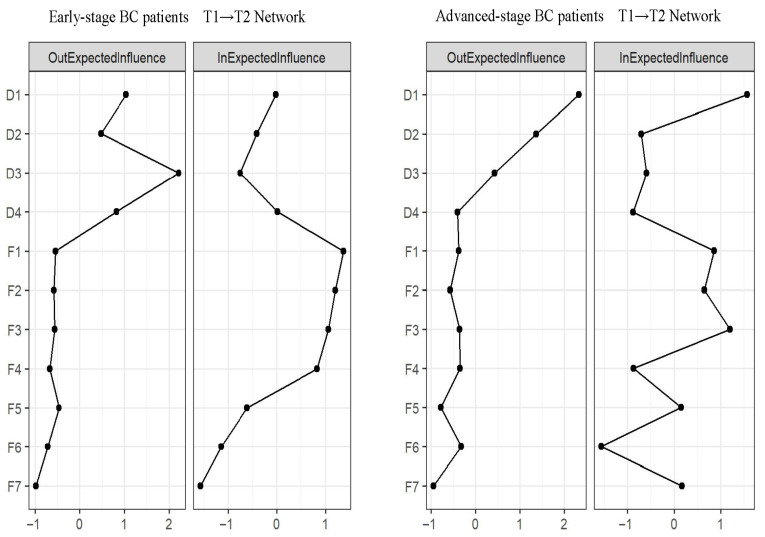
The comparison of symptom centrality estimates for T1 → T2 network.

Figure 3 and Figure 4 shown the details of the network comparison. Regarding the results for early-stage BC patients, the strongest cross-lagged association was D3: time awareness → F3: psychological distress (OR = 1.363). Additionally, the top out-EI and in-EI nodes were D3 (Time awareness, OEI = 2.214) and F1 (Triggers, IEI = 1.368). For advanced-stage BC patients, the strongest cross-lagged association was D2: Emotion → F7: Coping strategies (OR = 1.363). Both the top out-EI and in-EI nodes were D1 (Cognition, OEI = 2.334, IEI = 1.570). Further details can be seen in Tables S2 and S3, and Supplementary Figures S6 and S7.

## 4. Discussion

This study advances our understanding of the longitudinal relationship between DA and FCR symptoms in Chinese women with breast cancer, using cross-lagged panel network analysis to examine symptom dimensions and stage-specific differences prospectively over time.

While we believe this is the first application of CLPN to prospectively model the interconnections between DA and FCR in this specific population, prior studies have explored related aspects in other contexts. For instance, longitudinal research on FCR trajectories in Chinese newly diagnosed cancer patients (including breast cancer) has identified heterogeneous patterns influenced by factors like social support and coping styles [31]. Similarly, prospective studies in Western populations, such as those on head and neck cancer survivors, have tracked FCR courses and associated factors like metacognition [32]. Cross-sectional investigations have also linked DA to FCR in ovarian cancer patients [9] and general Western samples [3], suggesting predictive roles for DA in amplifying FCR. However, these studies often lack a network perspective or focus on non-Chinese cohorts. Our findings build on this body of work by revealing temporal predictive relationships specific to Chinese breast cancer women, highlighting the need for culturally tailored interventions.

Regarding the optimal intervention symptoms of DA and FCR, node D1 (cognition) emerged as the highest OEI symptom, suggesting that cognition during T1 strongly influences other symptoms during T2. The strongest cross-lagged edge was from D2 (emotion) to D1 (cognition) in terms of longitudinal processes within death anxiety. Specifically, cognition emerged as the pivotal symptom in the progression of other DA symptoms over time, and it was found to be strongly linked with emotional responses. In other words, the presence of emotion could act as a catalyst for the development of cognition. Similarly to previous research [33] focusing on DA problems, cognition and death-related emotions play a central role in maintaining the additive cycle of death anxiety. Emotion-focused coping strategies exerted an indirect influence on the level of DA in breast cancer patients, mediated through their perceptions of the disease [34]. Based on these understandings, this study extends existing findings by revealing the potential role of cognition and death-related emotions as the basis for negative reinforcement in death anxiety.

In addition, node F3 (psychological distress) was identified as the highest IEI symptom, meaning that it was the most affected by the other symptom nodes. That is, psychological distress is likely to worsen when some of the other symptoms in the network are aggravated, so this symptom also needs to be taken seriously. The strongest cross-lagged edge was from F2 (severity) to F1 (triggers) in terms of longitudinal processes within FCR. This means that the severity of FCR at the time of discharge would influence their triggers for FCR three months later. The possible reason is that the higher level of FCR after discharge from the hospital acts instead to make patients more worried about their condition, such as being more concerned about pain symptoms and frequency of review [35], thereby increasing sensitivity or awareness of daily trigger events [36]. Thus, it is particularly important to carry out disease education and targeted psychological intervention for breast cancer patients in a timely manner [37].

Regarding the progression over time in the interrelation between DA and FCR symptoms, D1 (cognition) was found to be the connecting symptom, bridging DA and FCR. The current model of cancer-related anxiety proposes that cognitive content (threat assessment and intrusion) would affect FCR [12]. It is also worth noting that discussing death is often a taboo [38] and cancer is almost considered as synonymous with death and untreated disease [39]. This cultural background shapes the cognitive component of DA [34], and the cognition of death could affect the degree of fear of cancer recurrence [3]. Thus, the influence of death-related cognition on both DA and FCR is of great importance, as well as the bridge between the two long-term coexisting among BC patients. On the other hand, the top strongest bridging edge was from D3 (time awareness) to F3 (psychological distress). According to a previous study [40], an individual’s response to a major life event is a gradual process of adaptation. BC patients may harbor a strong sense of their own finite lives at the time of discharge from the hospital, and this initial high level of anxiety, if not appropriately managed or alleviated, may persist in subsequent months, leading to long-term psychological distress.

In terms of network differences in patients with different stages of breast cancer, we additionally found that D3 (time awareness) in patients with early-stage breast cancer was significantly predictive of other network symptom nodes. Realizing the finite nature of life is a common human experience, and for patients with early-stage breast cancer, this realization appears to be heightened by the diagnosis of the disease and after discharge from the hospital [41]. For advanced-stage BC patients, both the top out-EI and in-EI nodes were D1 (cognition), and the strongest cross-lagged association was from D2 (emotion) to F7 (coping strategies). Danger Ideation Reduction Therapy (DIRT), led by cognitive restructuring [42], may be particularly effective in addressing the coexistence of DA and FCR in patients with advanced breast cancer. Previous studies had shown that coping strategies were associated with emotional outcomes [43]. Identifying the emotional issues associated with dying at discharge from advanced breast cancer could help improve their future coping strategies.

This study has some limitations. Firstly, although we based our network analysis on longitudinal data, the scope was limited to two collection points. Secondly, in terms of subjects, our study only included Chinese women with breast cancer. While our outcomes precisely encapsulate the characteristics of this group, further validation in populations with greater diversity could be warranted to ensure the generalizability of these findings. While our cross-lagged panel network analysis reveals directional predictive relationships, these do not establish strict causality, as unobserved confounders or bidirectional influences may exist. We interpret these as temporal associations that highlight potential pathways for intervention. Future randomized controlled trials (RCTs) are recommended to test whether targeting DA (e.g., via existential therapy) can causally reduce FCR, confirming the clinical utility of our identified interconnections. Our two-time point design captures short-term dynamics; future studies with additional follow-ups could assess persistence. Finally, this study performed a network comparison of cancer stages in BC patients after a binary classification. Although this dichotomy process makes it easier to explain and analyze, it could oversimplify complex psychological phenomena.

Our study emphasized the practical value of cross-lagged network models in exploring the relationship between DA and FCR. From a theoretical point of view, it highlighted the phenomenon of comorbidity between DA symptoms and FCR symptoms that progresses over time. In practical terms, the links between bridge symptoms could be disrupted to prevent the intensification of intensive connections between comorbid mental health problems [44]. We also demonstrated how death cognition had effects on and further predicted other symptoms, and the emotion evoked by death anxiety and the severity of FCR created a negative cycle interconnecting these two psychological concerns. In addition, there were differences in the optimal intervention targets for breast cancer patients in different tumor stages, and the reasons need to be further explored in the future.

## 5. Conclusions

Considering that death anxiety co-occurs with fear of cancer recurrence in breast cancer patients, patients’ death-related cognition and emotional regulation of death may be the best target for further interventions. Identifying the differences in death cognition of breast cancer patients in different tumor stages, improving coping skills of emotional distress, and good mental nursing may be powerful measures to improve the coexistence condition of DA and FCR.

## Figures and Tables

**Table 1:** Demographic characteristics of the sample (n = 426).

Variable	Classification	Total [n (%)]
Age (years)		
	18~44	173 (40.61)
	45~59	210 (49.30)
	≥60	43 (10.09)
Education		
	Junior secondary and less	218 (51.18)
	High school/junior college	140 (32.86)
	Bachelor and more	68 (15.96)
Marital status		
	Married	382 (89.67)
	Unmarried/Divorced/widowed	44 (10.33)
Occupation		
	In-service	350 (82.16)
	No occupation	34 (7.98)
	Retirement	42 (9.86)
Household income (RMB)		
	<3000	134 (31.46)
	3000~5999	141 (33.09)
	≥6000	151 (35.45)
Cancer stage		
	I	44 (10.33)
	II	173 (40.61)
	III	168 (39.44)
	VII	41 (9.62)

**Table 2:** Subscale labels and descriptive statistics at two time points (n = 426).

Subscales	Label	Time Point 1		Time Point 2	
		M	SD	M	SD
Cognition	D1	3.30	2.13	2.58	1.74
Emotion	D2	2.23	1.45	2.07	1.59
Time awareness	D3	1.41	0.82	0.73	0.75
Stress and pain	D4	1.98	1.18	1.19	1.23
Triggers	F1	14.67	6.66	13.13	5.76
Severity	F2	17.40	5.34	15.37	4.68
Psychological distress	F3	7.05	2.95	5.23	2.28
Functioning impairment	F4	13.58	4.53	8.93	5.41
Insight	F5	5.84	2.46	9.11	4.68
Reassurance	F6	8.58	3.20	7.24	3.07
Coping strategies	F7	17.31	7.20	15.53	9.18

**Table 3:** Adjacency matrix of the T1 → T2 cross-lagged panel network. Independent variables (i.e., predictors) are in rows, and dependent variables are in columns.

	D1	D2	D3	D4	F1	F2	F3	F4	F5	F6	F7
**D1**	1.000	1.142	1.048	1.000	1.085	**1.269**	1.192	1.293	1.169	1.105	1.258
**D2**	**1.332**	1.000	1.000	1.109	1.091	1.000	1.014	1.000	1.000	1.020	1.000
**D3**	1.000	1.000	1.000	**1.225**	1.048	1.198	**1.363**	1.151	1.041	1.000	0.992
**D4**	1.122	1.037	1.131	1.000	1.175	1.000	1.000	1.000	1.000	0.919	1.000
**F1**	1.000	1.000	1.000	0.990	1.000	1.000	0.976	0.972	1.000	**1.028**	1.000
**F2**	1.000	1.000	1.012	1.000	**1.063**	1.000	0.978	0.949	0.981	0.947	0.896
**F3**	1.000	1.000	1.000	**1.068**	1.000	1.000	1.000	1.000	1.000	0.956	0.960
**F4**	1.000	1.000	1.000	1.000	1.000	1.000	1.000	1.000	1.000	0.983	0.941
**F5**	1.000	1.000	1.000	0.978	1.000	1.000	1.000	1.000	1.000	1.005	0.942
**F6**	0.967	1.000	0.977	0.989	1.000	1.000	1.011	1.000	1.022	1.000	1.000
**F7**	1.000	1.000	1.000	0.981	0.929	0.944	0.928	0.920	0.943	0.979	1.000

**Note:** D1: Cognition; D2: Emotion; D3: Time awareness; D4: Stress and pain; F1: Triggers; F2: Severity; F3: Psychological distress; F4: Functioning impairment; F5: Insight; F6: Reassurance; F7: Coping strategies.

## Data Availability

The datasets generated during and/or analyzed during the current study are not publicly available due to participants’ privacy but are available from the corresponding author on reasonable request.

## Supplementary Materials

The following supporting information can be downloaded at https://www.mdpi.com/article/10.3390/curroncol32120685/s1: Figure S1: The power analysis simulation results of T1 → T2 network; Figure S2: Stability of centrality measures in network; Figure S3: Bootstrapped 95% confidence intervals around each edge weight for network using T1 to predict T2. Red lines indicate the edge weight in the estimated sample network; Figures S4 and S5: Edge weight difference tests for network. Black boxes indicate edges that significantly differ from each other (*p* < 0.05), and gray boxes indicate edges that do not significantly differ; Centrality difference tests for the network. Black boxes indicate symptoms that significantly differ in centrality (*p* < 0.05), and gray boxes indicate symptoms whose centrality does not significantly differ; The comparison of stability of centrality measures for T1 → T2 networks; Figures S6 and S7: The comparison of edge weight difference tests for T1 → T2 network. Table S1. Correlation analysis between death anxiety and fear of cancer recurrence at T1; Table S2. Adjacency matrix of the T1 → T2 cross-lagged panel network among early-stage BC patients. Independent variables (i.e., predictors) are in rows, and dependent variables are in columns; Table S3. Adjacency matrix of the T1 → T2 cross-lagged panel network among advanced-stage BC patients. Independent variables (i.e., predictors) are in rows, and dependent variables are in columns.

## Abbreviations

The following abbreviations are used in this manuscript:

DA	Death anxiety
FCR	Fear of cancer recurrence
CLPN	Cross-lagged panel networks
DIRT	Danger ideation reduction therapy
