# Supplementary material for: Predictive Relationships Between Death Anxiety and Fear of Cancer Recurrence in Patients with Breast Cancer: A Cross-Lagged Panel Network Analysis
Source: Curr Oncol. 2025 Dec 3;32(12):685. doi: 10.3390/curroncol32120685 (PMC12731887; doi:10.3390/curroncol32120685)
Supplement: Supplementary file 1 [file curroncol-32-00685-s001.zip › Supplementary Tables.pdf]

Table S1. Correlation analysis between death anxiety and fear of cancer recurrence at T1.

	D1	D2	D3	D4	F1	F2	F3	F4	F5	F6	F7
D1	1										
D2	0.821**	1									
D3	0.572**	0.587**	1								
D4	0.501**	0.495**	0.433**	1							
F1	0.379**	0.344**	0.300**	0.362**	1						
F2	0.385**	0.339**	0.282**	0.299**	0.832**	1					
F3	0.422**	0.403**	0.276**	0.242**	0.612**	0.679**	1				
F4	0.367**	0.343**	0.268**	0.258**	0.621**	0.663**	0.772**	1			
F5	0.473**	0.431**	0.312**	0.313**	0.648**	0.695**	0.738**	0.730**	1		
F6	0.368**	0.320**	0.279**	0.265**	0.426**	0.441**	0.451**	0.424**	0.552**	1	
F7	0.276**	0.312**	0.284**	0.186**	0.498**	0.434**	0.282**	0.351**	0.323**	0.284**	1

Note: ***P*<0.001. D1: Cognition; D2: Emotion; D3: Time awareness; D4: Stress and pain; F1: Triggers; F2: Severity; F3: Psychological distress; F4: Functioning impairment; F5: Insight; F6: Reassurance; F7: Coping strategies

Table S2. Adjacency matrix of the T1 → T2 cross-lagged panel network among early-stage BC patients. Independent variables (i.e., predictors) are in rows, and dependent variables are in columns.

	D1	D2	D3	D4	F1	F2	F3	F4	F5	F6	F7
D1	1.000	1.234	1.017	1.000	1.000	1.182	1.066	1.180	1.091	1.022	1.128
D2	1.199	1.000	1.000	1.063	1.288	1.000	1.040	1.000	1.000	1.000	1.000
D3	1.115	1.000	1.000	1.252	1.000	1.286	1.465	1.427	1.063	1.000	1.000
D4	1.027	1.000	1.093	1.000	1.328	1.178	1.078	1.035	1.057	1.000	1.000
F1	1.000	1.000	1.000	0.992	1.000	1.000	1.000	1.000	1.000	1.000	1.000
F2	1.000	1.015	1.013	1.000	1.091	1.000	0.947	0.933	0.994	1.000	0.977
F3	1.061	1.000	1.000	1.049	1.000	1.000	1.000	1.000	0.981	1.000	0.891
F4	1.000	1.000	1.000	1.000	1.000	1.000	1.000	1.000	1.000	1.000	0.918
F5	1.000	1.000	1.000	1.000	1.000	1.010	1.022	1.010	1.000	1.000	1.000
F6	0.906	0.960	1.000	0.970	1.000	1.000	1.034	1.000	1.024	1.000	1.000
F7	1.000	1.000	1.000	0.992	0.955	0.964	0.934	0.941	0.951	1.000	1.000

Table S3. Adjacency matrix of the T1 → T2 cross-lagged panel network among advanced-stage BC patients. Independent variables (i.e., predictors) are in rows, and dependent variables are in columns.

	**D1**	**D2**	**D3**	**D4**	**F1**	**F2**	**F3**	**F4**	**F5**	**F6**	**F7**
D1	1.000	1.027	1.040	1.063	1.234	1.314	1.209	1.293	1.228	1.107	1.294
D2	1.336	1.000	1.000	1.008	1.000	1.000	1.176	1.000	1.101	1.194	**1.333**
D3	1.000	1.000	1.000	1.018	1.165	1.087	1.261	1.000	1.000	0.976	1.000
D4	1.140	1.074	1.096	1.000	1.000	1.000	0.899	0.935	0.977	0.847	0.970
F1	1.000	1.000	1.000	1.000	1.000	1.000	0.958	0.968	1.000	1.032	1.000
F2	1.000	1.000	1.000	1.000	1.033	1.000	0.991	0.973	0.989	0.945	0.900
F3	1.000	1.000	1.000	1.000	1.000	1.000	1.000	1.000	1.000	0.972	1.000
F4	1.000	1.000	1.000	1.000	1.000	1.000	1.000	1.000	1.000	0.981	0.995
F5	1.000	1.000	1.000	1.000	1.000	1.000	1.000	0.998	1.000	0.931	0.753
F6	1.000	1.000	0.984	1.000	1.000	1.000	1.000	1.000	1.012	1.000	1.000
F7	1.000	1.000	1.000	0.983	0.929	0.924	0.922	0.905	0.935	0.972	1.000
