# Supplementary figures and images for: Predictive Relationships Between Death Anxiety and Fear of Cancer Recurrence in Patients with Breast Cancer: A Cross-Lagged Panel Network Analysis
Source: Curr Oncol. 2025 Dec 3;32(12):685. doi: 10.3390/curroncol32120685 (PMC12731887; doi:10.3390/curroncol32120685)

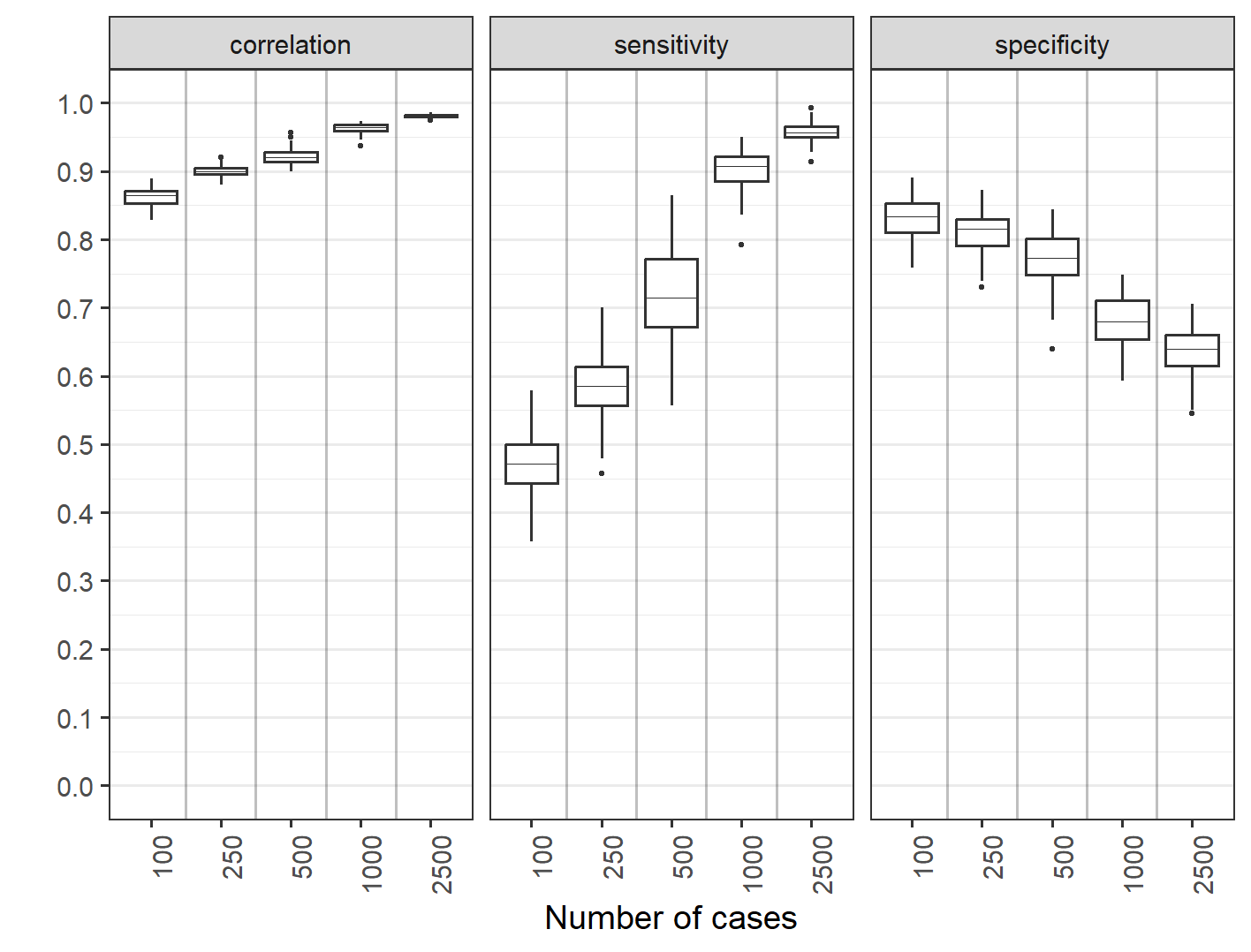

Supplement: Supplementary file 1 [file curroncol-32-00685-s001.zip › Supplementary Figure S1.png]

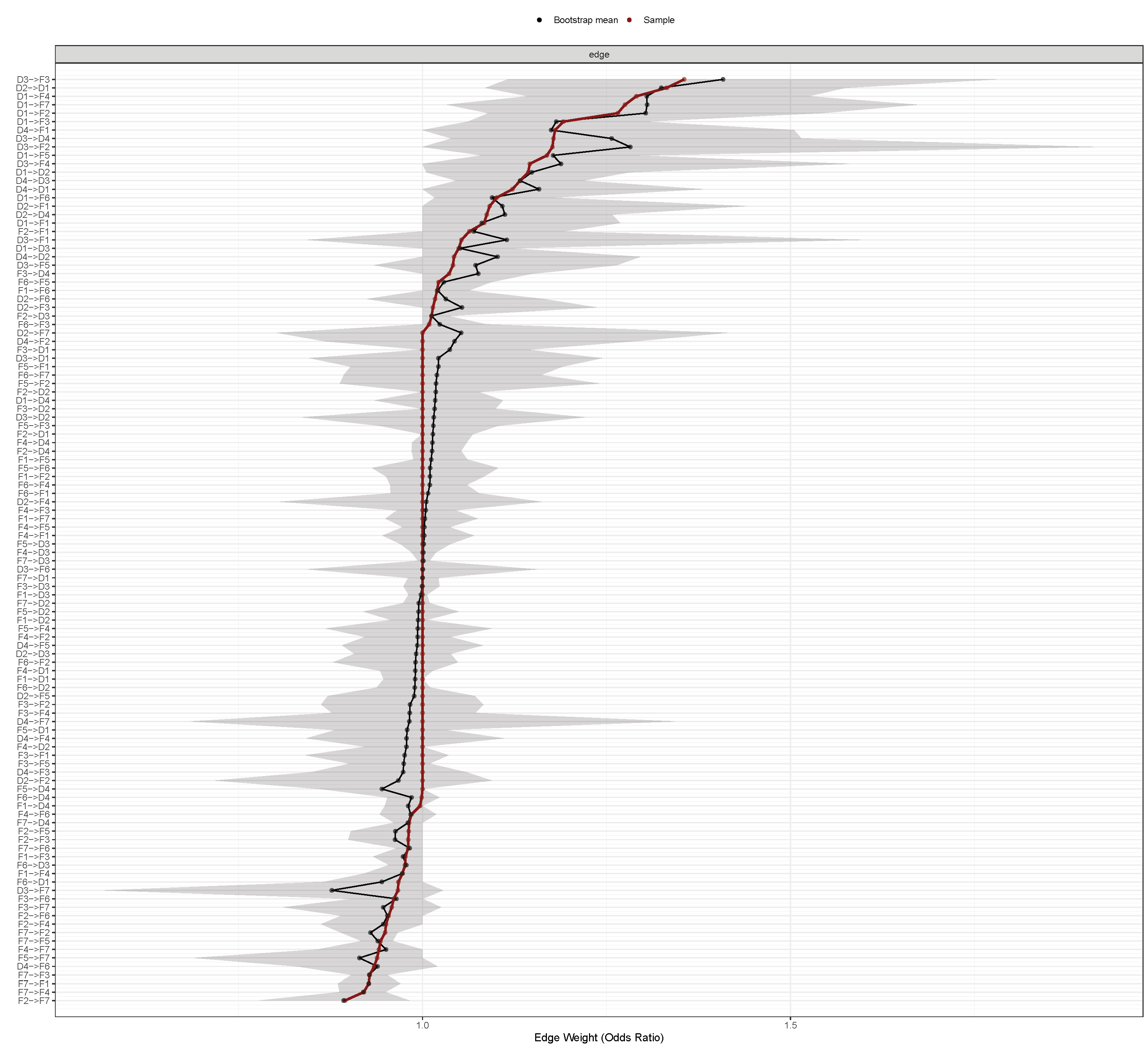

Supplement: Supplementary file 1 [file curroncol-32-00685-s001.zip › Supplementary Figure S2.png]

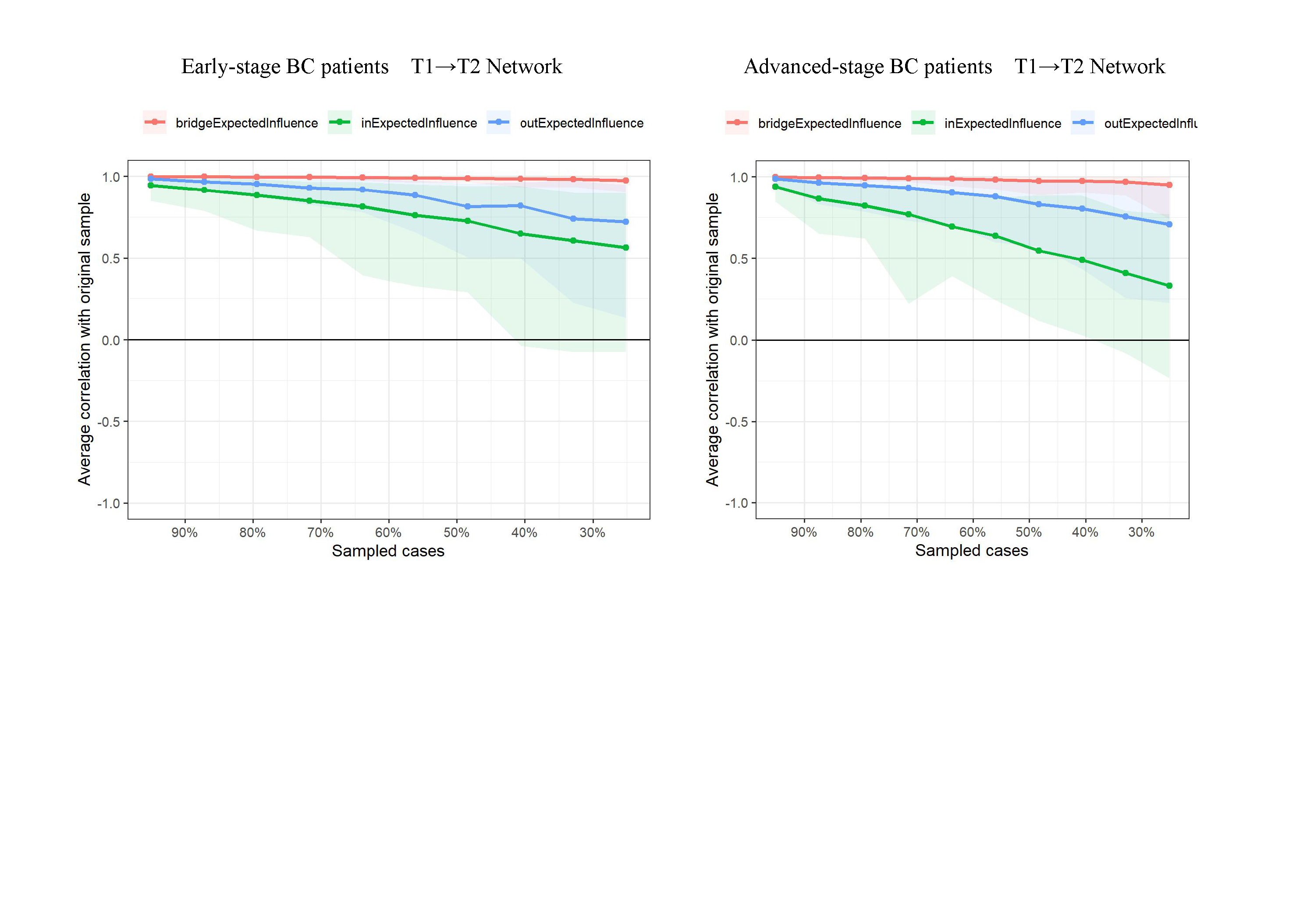

Supplement: Supplementary file 1 [file curroncol-32-00685-s001.zip › Supplementary Figure S6.png]

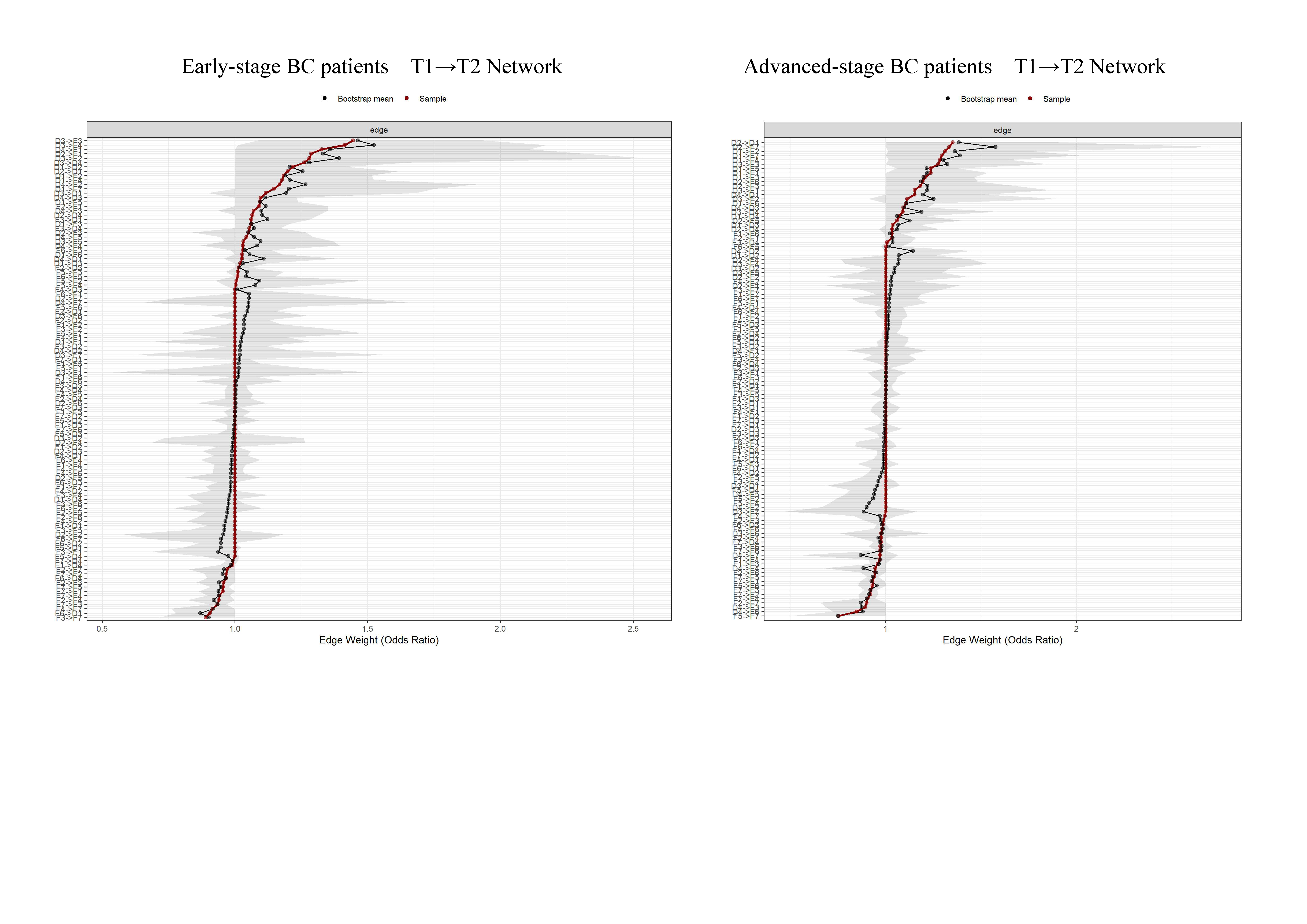

Supplement: Supplementary file 1 [file curroncol-32-00685-s001.zip › Supplementary Figure S7.png]

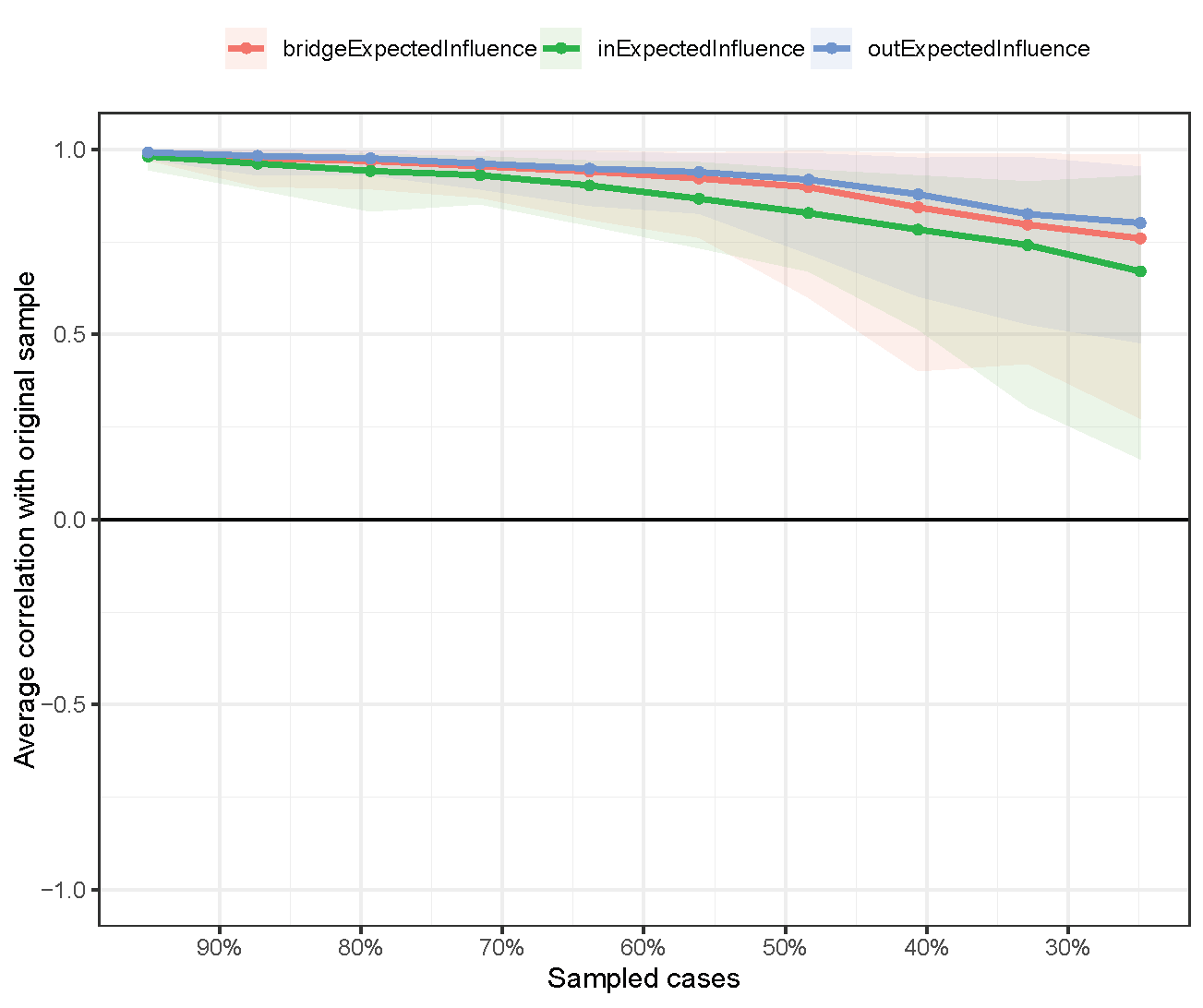

Supplement: Supplementary file 1 [file curroncol-32-00685-s001.zip › Supplementary Figure S3.png]

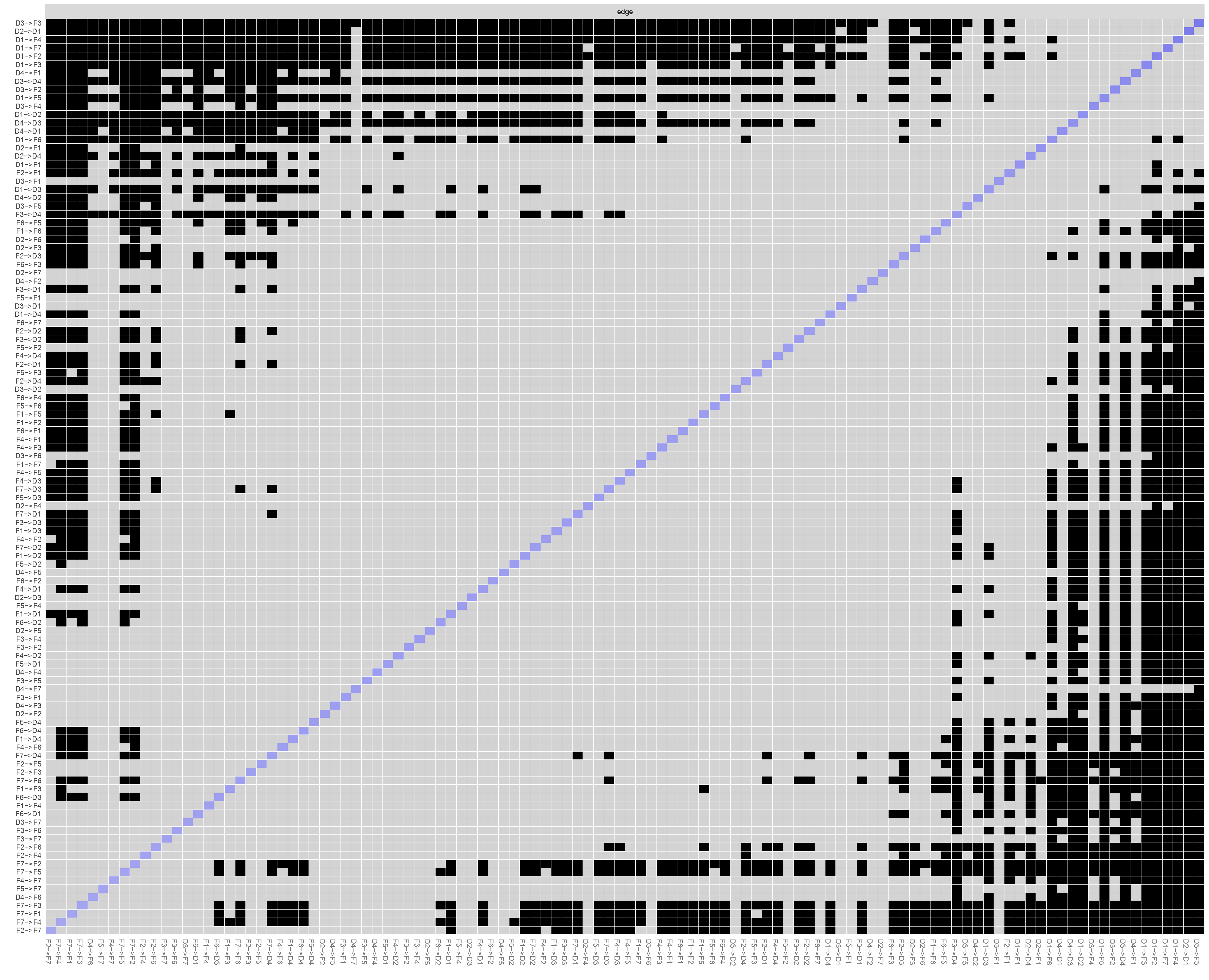

Supplement: Supplementary file 1 [file curroncol-32-00685-s001.zip › Supplementary Figure S4.png]

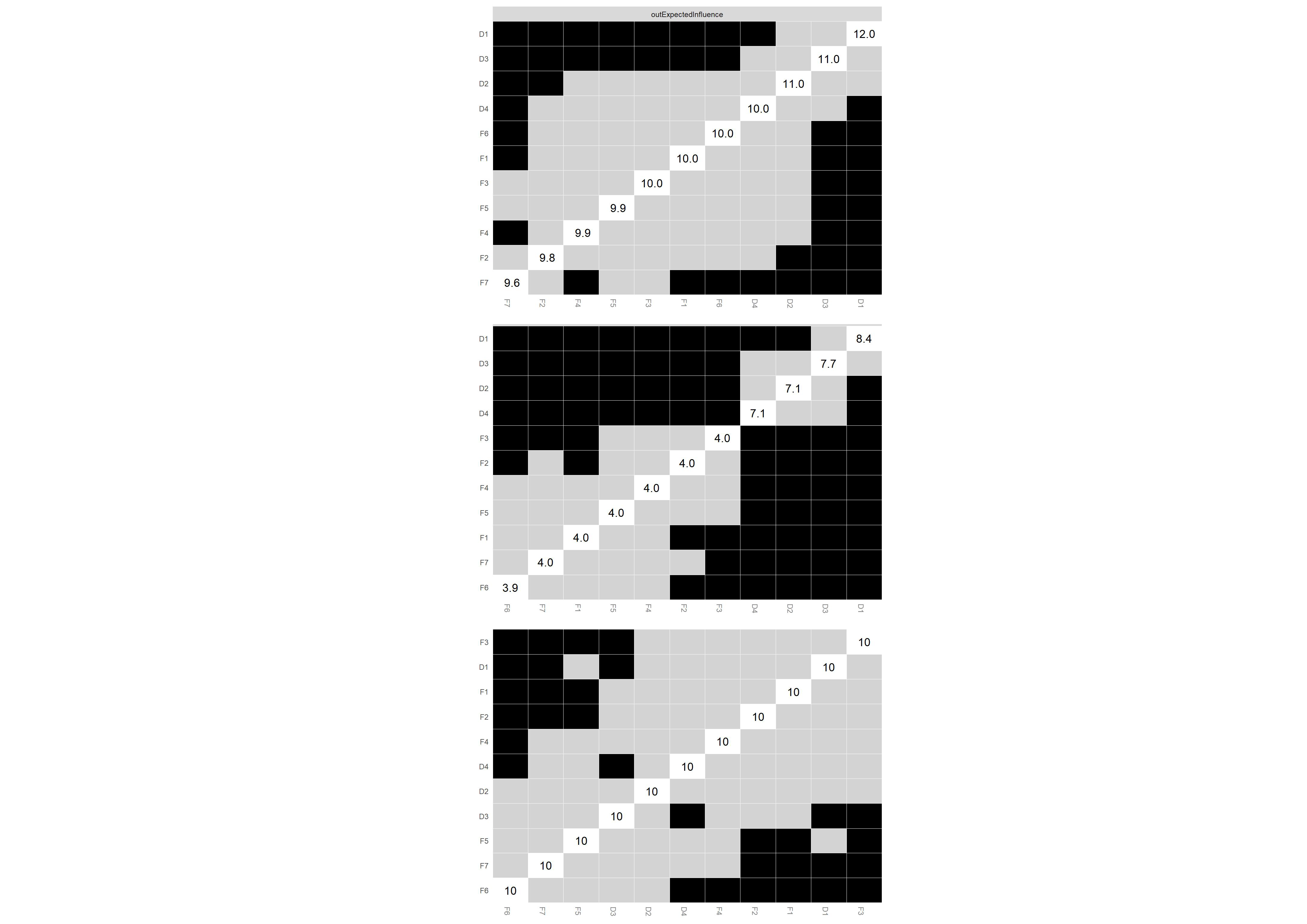

Supplement: Supplementary file 1 [file curroncol-32-00685-s001.zip › Supplementary Figure S5.png]
